# Genetic and Environmental Determinants of T Helper 17 Pathogenicity in Spondyloarthropathies

**DOI:** 10.3389/fgene.2021.703242

**Published:** 2021-09-22

**Authors:** Davide Simone, Alessia Stingo, Francesco Ciccia

**Affiliations:** Dipartimento di Medicina di Precisione, Section of RheumatologyUniversità degli Studi della Campania L. Vanvitelli, Naples, Italy

**Keywords:** T helper 17 cell, Spondyloarthropathies, ankylosing spondylitis, genetic risk, genome-wide association studies, interleukin 17, pathogenicity

## Abstract

In Spondyloarthropathies (SpA), a common group of immune-mediated diseases characterised by excessive inflammation of musculo-skeletal structures and extra-articular organs, T helper 17 (Th17) cells are widely considered the main drivers of the disease. Th17 are able to modulate their genes according to the immune environment: upon differentiation, they can adopt either housekeeping, anti-bacterial gene modules or inflammatory, pathogenic functions, and only the latter would mediate immune diseases, such as SpA. Experimental work aimed at characterising Th17 heterogeneity is largely performed on murine cells, for which the *in vitro* conditions conferring pathogenic potential have been identified and replicated. Interestingly, Th17 recognising different microorganisms are able to acquire specific cytokine signatures. An emerging area of research associates this heterogeneity to the preferential metabolic needs of the cell. In summary, the tissue environment could be determinant for the acquisition of pathogenetic features; this is particularly important at barrier sites, such as the intestine, considered one of the key target organs in SpA, and likely a site of immunological changes that initiate the disease. In this review, we briefly summarise genetic, environmental and metabolic factors that could explain how homeostatic, anti-microbial Th17 could turn into disease-causing cells in Spondyloarthritis.

## Introduction

T helper lymphocytes, characterised by the expression of CD4 on their surface, are the central cell subset of adaptive immunity. They are able to recognise protein antigens belonging to microorganisms thanks to a unique receptor expressed on their surface [T-cell receptor (TCR)] and shape an organised response against them. When a self-antigen is erroneously recognised, or when the activation threshold is altered, CD4+ T cells can cause pathological responses, characterised by uncontrolled inflammation. Together with the recognition of the antigen and the TCR engagement signal, integrated stimuli dictate the transcriptional changes that guide the differentiation of the naïve T cell towards a specialised function. These stimuli include cytokines, soluble mediators or bacterial products in the microenvironment. Specific intracellular pathways, including Stat (signal transducer and activator of transcription) proteins, regulate this process, which eventually leads to the induction of a dominant transcription factor (TF). The lineage-specific (‘master’) TF controls the transcriptional programme of the cell, including specific cytokine production and the expression of chemokine receptors that mediate trafficking to the organs: this network helps each T-cell subset to exert specific functions in response to antigens, and in the tissues. The central TFs are as: T-box protein expressed in T cells (T-bet) in Th1, GATA-binding protein 3 in Th2, retinoic acid-related orphan receptor gamma-t (ROR-ɣt) in T helper 17 (Th17) and Forkhead box P3 in Tregs (Zheng and Flavell, 1997; Szabo et al., 2000; Fontenot et al., 2003; Harrington et al., 2005).

Dysregulated mechanisms in various steps of T-cell commitment, maturation and response to challenges can contribute to pathogenic responses, such as immune-mediated conditions for Th1 and Th17 cells, and allergic responses for Th2 cells. Among immune-mediated diseases driven by Th17 cells are Spondyloarthropathies (SpA), a group of inflammatory arthritides, including Ankylosing Spondylitis (AS) and Psoriatic Arthritis (PsA), characterised by inflammation and structural damage of several musculo-skeletal structures and organs. In these diseases, genetic and immunological alterations suggest a dysregulated ‘type 17’ response (Simone et al., 2018), that is the arm of cellular immunity characterised by the production of IL-17 and orchestrated by Th17. The focus of this short review will be on Th17 cells, and the genetic and environmental mechanisms that likely determine their pathogenic behaviour thought to cause SpA.

## Th17 Differentiation

**Figure 1:**
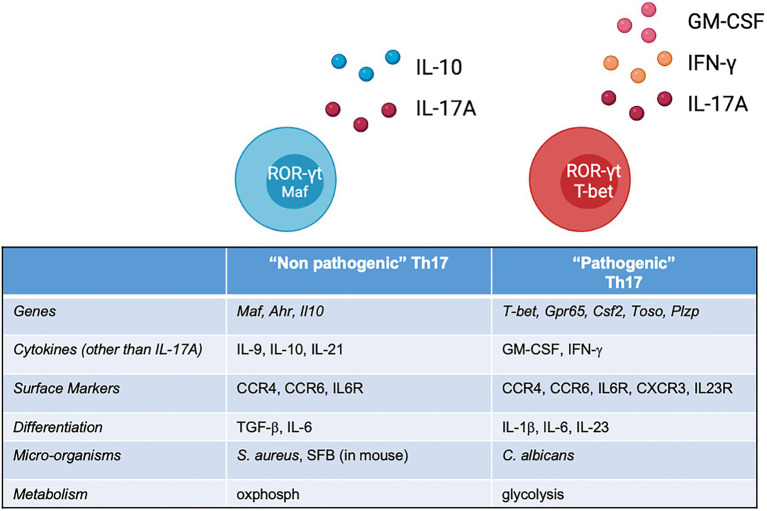
List of features of ‘non-pathogenic’ and ‘pathogenic’ Th17 cells identified across several studies, most of which performed on murine cells. *In vivo*, this classification should not be considered bimodal, but rather a continuum of states, shaped by the local environment.

The cytokine environment provides an important contribution to the T naïve fate decision. Although being a disputed matter for years, owing to between-species differences and experimental settings, it is accepted that the process of differentiation towards Th17 requires the presence of IL-6, IL-1β and variable concentrations of TGF-β (Bettelli et al., 2006; Veldhoen et al., 2006; Zhou et al., 2007). The IL-6 receptor, in particular, activates the JAK1/STAT3 pathway to induce the lineage defining TF ROR-ɣt (Yang et al., 2007). The STAT3/ROR-ɣt axis is the cornerstone of Th17 differentiation, but more TFs come into play in the process, such as RORa, Ahr, IRF4 and BATF (Brüstle et al., 2007; Schraml et al., 2009), with the last two possibly induced even before ROR-ɣt, characterising a sort of ‘pre-Th17’ status (Ciofani et al., 2012). These markers set the initial chromatin accessibility that allows a transcriptional programme, further defined by ROR-ɣt and decisive for the expression of effector genes (*Il17a* and *Il17f*). As multiple players are involved in the differentiation at different stages, the previously accepted paradigm of T helper differentiation as a linear, irreversible process is not actual. As demonstrated by knock down experiments, inactivation of these regulatory nodes leaves space to transcriptional instability and plasticity: in this scenario, the cytokine milieu could favour, or inhibit, molecular determinants at various stages of the differentiation phase, or even divert the programme towards other T helper lineage. TGF-β is a paradigmatic case: initially identified as essential for murine Th17 differentiation (Mangan et al., 2006), but not for human Th17 cells (Acosta-Rodriguez et al., 2007), and subsequently reinstated as necessary for both human and murine differentiation (Manel et al., 2008; Volpe et al., 2008). In practice, according to a parallel interpretation, two different cytokine cocktails lead to two different Th17 ‘flavours’: TGF-β, together with IL-6, induces ‘non-pathogenic’ Th17 cells characterised by the co-expression of IL-10 (McGeachy et al., 2007); on the other hand, IL-6 and IL-23 (and no TGF-β) lead to differentiation into ‘pathogenic’ Th17 cells (Ghoreschi et al., 2010; Figure 1). Both subsets would express ROR-ɣt, but ‘pathogenic’ Th17 cells, more plastic and polymorphic, have a tendency to transition towards Th1, and consequently the production of IFN-ɣ. This distinction, popularised by studies that use *in vitro* differentiation assays of murine Th17, likely does not find strict correspondence *in vivo*. In experimental autoimmune encephalomyelitis (EAE), Th17 cells grown in presence of TGF-β are, in fact, also pathogenic (Kebir et al., 2007). It is also difficult to conceive conditions completely devoid of TGF-β, very common in plasma and tissues *in vivo*, and often present (in active or inactive form) in the serum enriching *in vitro* culture media. What might be crucial is the concentration, and the gradient, of TGF-β: high concentrations of TGF-β induce Treg-associated genes while restraining T-bet and other Th1 genes and possibly inhibit Th17 pathogenic responses. The developmental overlap between Th1, Th17 and Treg might in fact be caused by complex cytokine dynamics. At the opposite side of the spectrum, IL-23 has long been considered the main ingredient for pathogenic differentiation and the initiator of the pro-inflammatory module.

## Th17 and Spondyloarthropathies

There is considerable evidence that indicates a central role of Th17 cells in the pathogenesis of SpA. The proportion of Th17 is higher in the blood of AS patients (Shen et al., 2009) and so are the serum concentrations of IL-17A, as reported in a number of studies by different groups (reviewed in Taams et al., 2018). In addition to experimental evidences, current clinical practice offers the empirical demonstration of the importance of ‘type 17’ lymphocytes in the pathogenesis of SpA: secukinumab (an anti-IL-17A monoclonal antibody) is currently widely used in AS and PsA, while a number of novel agents against IL-17 superfamily cytokines are in the final experimental stages or about to be licenced for clinical use (van der Heijde et al., 2018, 2020).

An important series of evidences originates from genetic studies: Genome-Wide Association Studies (GWAS; International Genetics of Ankylosing Spondylitis Consortium (IGAS) et al., 2013) have convincingly established the association between AS and genetic variants in loci involved in the Th17 pathway, including *TYK2*, *IL6R*, *IL1R1*, *IL1R2* and *IL23R*, whose products are important for the induction and survival of the Th17 response. In particular, the existence of a genetic variant of the IL-23 receptor gene, possibly causing the altered signalling predisposing to AS, emerged early from one of the first association studies (Wellcome Trust Case Control Consortium et al., 2007). Although one AS-associated SNP (rs11209026, also seen in psoriasis and IBD) causes a missense variation that alters IL-23R signalling, impairing Th17 responses (Di Meglio et al., 2011, 2013), the effect of the other gene variants is unclear. Recently, epigenetic data on chromatin remodelling and transcription factor (TF) binding helped to characterise a second independently AS-associated SNP located in the IL23R-IL12RB2 intergenic region. The observed higher number of IFN-g-secreting cells in the risk allele carriers (Roberts et al., 2016) could in fact explain the functional association with the disease. Although this work could not confirm this alteration is present on Th17 specifically, it provides an example of how allelic variations affect the genetic/epigenetic regulation of inflammatory pathways, thus potentially induce pathogenic functions in Th17 cells. Other genes, whose variants associate with AS, have been carefully studied in the search of an alteration of Th17 responses that can cause AS. *STAT3* and *TYK2*, for example are mediators of Th17 immunity. Their protein products play a central role in IL-6 and IL-23 signalling: they are activated by phosphorylation and indirectly act as transcriptional activators of Th17 differentiation, *via* a chain of other intermediates. Several SNPs within *STAT3* locus have been associated to AS and CD (Danoy et al., 2010), but their impact on Th17 function in these conditions has never been clarified, while different causing SNPs of *STAT3* showed to impact Th17 differentiation in other autoimmune diseases (Tripathi et al., 2017). Another important GWAS hit, involving a gene involved in Th17 responses, is *TYK2*. The AS-associated SNPs (rs12720356) at the *TYK2* locus were found to be associated with increased Th1 frequency and AS disease progression: this, together with the evidence that Tyk2 inhibition is an effective strategy in the experimental model of AS (Gracey et al., 2020), makes the search for functional variants of genes that orchestrate the Th17 response a biologically relevant enterprise, that is also very promising from a translational point of view.

## Determinants of Th17 Pathogenicity

IL-23, a heterodimeric cytokine, is closely related to IL-12, with whom it shares one of the two subunits. Consistent with its kinship to IL-12, it is able to induce both IL-17 and IFN-γ (Aggarwal et al., 2003) in CD4+ cells. More precisely, it is dispensable for Th17 development, but it enhances an accessory transcriptional module, thought to be pathogenic (Volpe et al., 2008). Indeed, IL-23 persistence induces Th17 to acquire a Th1-like phenotype (Lee et al., 2009), and lack of IL-23R on T cells prevents experimental colitis (Ahern et al., 2010). IL-23, together with IL-1, has been associated with Th17 in autoimmune models (Cua et al., 2003; Langrish et al., 2005; McGeachy et al., 2009). In particular, IL-23 is the key ingredient of pathogenicity in EAE because it induces high levels of T-bet, IL-23R and GM-CSF in the Th17 cell (Lee et al., 2012). Importantly, IL-23R is not constitutively expressed on the naïve T cell, but it appears during the Th17 differentiation secondarily to IL-6 signalling (Chung et al., 2009; Ghoreschi et al., 2010). Through this and other feed forward mechanisms (Kishi et al., 2016; Meyer Zu Horste et al., 2016), the Th17 inflammatory programme is stabilised.

The aforementioned association of genetic variants of the IL23R gene region with AS (International Genetics of Ankylosing Spondylitis Consortium (IGAS) et al., 2013) led to hypothesise that IL-23 was the main driver for pathogenic responses in AS as well. Early experimental data in humans did not confirm this: IL-23 blockade proved ineffective on the axial symptoms in AS (Baeten et al., 2018). Few hypotheses have been proposed to explain this: IL-23 might still be a key player in the initiation phase, losing importance when the disease is already established. An alternative explanation postulates the presence of other IL-23-independent drivers of Th17 pathogenicity. In reality, immune cytokine ‘axes’ (such as IL-23/17) are almost never linear: IL-23 and IL-17 do participate in the same branch of the immune response but at the same time, have distinct biology and affect different cell subsets in different tissues. Regardless of its induction, we now know that the effector functions of the Th17 pathogenic module consist in IL-17A plus additional inflammatory cytokines, such as IFN-ɣ and GM-CSF, the latter an emerging Th17-associated player of autoimmunity (El-Behi et al., 2011). Recent translational approaches in SpA are focusing on novel cytokine targets, such as GM-CSF (Al-Mossawi et al., 2017; Wade et al., 2019), or upstream pro-inflammatory intracellular signals, such as JAK/STAT (Hammitzsch et al., 2018), a strategy that would simultaneously regulate cell differentiation and inhibit multiple inflammatory cytokines.

Other genetic events leading to unrestrained, disease-causing differentiation are still not clear: *ex vivo* single-cell sequencing led to identify novel pathogenic genes (*Gpr65*, *Toso* and *Plzp*) promoting Th17-mediated CNS inflammation in EAE mice (Gaublomme et al., 2015; Wang et al., 2015), but none of which, as of today, have been validated in humans, with the partial exception of GPR65, an important TF in human GM-CSF + Th17 (Al-Mossawi et al., 2017). The gene, a GWAS hit associated with AS, encodes for a G-protein coupled receptor with an extracellular proton sensing domain, and strongly characterises GM-CSF-producing Th17, where its activation likely concurs to the production of said cytokine in the acidic environment, expanding the inflammatory potential of the Th17 cell.

## Th17 Response To Microbiota

Not only cytokines, but also different microorganisms have the ability to prime distinct Th17 responses (McGeachy and McSorley, 2012). A study in humans showed, through *in vitro* experiments, that Th17 cells primed with different pathogens are able to acquire specific cytokine signatures (Zielinski et al., 2012). Th17 are inherently plastic having evolved to tailor their response to different pathogens at different anatomical sites: one well-characterised example is provided by a work that described how gut-resident Th17 cells in mice, primed by segmented filamentous bacteria (SFB), exhibit little plasticity and are not involved in tissue inflammation, while Th17 induced by *C. rodentium* have pathogenic potential, and a preferential glycolytic metabolism (Omenetti et al., 2019). Our interpretation is that the combination of host features (e.g., presence of predisposing genetic variants and HLA haplotype) associated with either commensal or pathogenic microbiome would dictate the characteristics of Th17 responses to bacteria and fungi. A recent paper hypothesised that airway Th17 cells in airway inflammation, e.g., during acute allergic bronchopulmonary aspergillosis, could all be cross-reactive to commensal *Candida* (Bacher et al., 2019): it is conceivable that a similar phenomenon of cross-reactivity could be observed in Th17 from SpA patients in response to gut or skin microbes. At the moment, it is not clear whether Th17 cells driving the manifestations of SpA are primed in the barrier organs, such as gut or skin (Gracey et al., 2019). Certainly, specific microbe-induced experimental models, or even gnotobiotic animals, could help replicating different modes of Th17 induction in various disease models and highlight both pathogenic mechanism and natural regulatory responses, such as in the case of cMaf + ROR-ɣt+Tregs, a cell subset specialised in restraining pathogenic bacteria-induced Th17s (Xu et al., 2018).

As anticipated, emerging features of T-cell biology are their inherent instability (loss of expression of transcriptional signature) and plasticity (acquisition of TFs or cytokines typical of other lineages). They are often being seen upon *in vitro* restimulation (Hirota et al., 2011), but also *in vivo*: Th17 cells can acquire Th1-features and transdifferentiate into the so-called Th17.1 cells, a subset that is particularly evident in the inflammatory environment, such as the synovial fluid in the course of arthritis (Nistala et al., 2010). Conversely, Th17 can lose pathogenic features, or even transdifferentiate into Treg-like cells: under pro-inflammatory conditions in the gut, intestinal Th17 cells can differentiate into IL-10-producing Tr1-like cells, important in the resolution of inflammation (Gagliani et al., 2015). This aspect of Th17 biology can provide therapeutic opportunities, through the induction of an anti-inflammatory fate in former pathogenic Th17 cells.

## Th17 in Tissue Homeostasis

In the steady state, the natural role of Th17 cells is to maintain tissue homeostasis, by maintaining barrier integrity (Lee et al., 2015) and anti-microbial functions, in particular vs. pathogenic microbes, such as, in humans, *Candida* and *Salmonella* (McGeachy and McSorley, 2012). In mice, colonisation with the commensal SFB (Ivanov et al., 2009) is sufficient to provide Th17-mediated resistance to the intestinal pathogen *Citrobacter rodentium*.

These gut-specific Th17 cells with a homeostatic role can be defined as ‘non-pathogenic Th17 cells’ being largely responsive to microbial or food-derived antigens and not involved in inflammatory mediated diseases. However, additional environmental cues, such as colonisation by specific microbial species, genetic predispositions and toxins, alter the equilibrium by enforcing effector profiles of autoimmune Th17 cells that can cause pathogenic responses that could then drive disease in the musculo-skeletal structures. The possible driving role of intestinal immunity in the pathogenesis of AS is indirectly confirmed by the susceptibility genes for Crohn’s disease (CD), a form of inflammatory bowel disease akin to the colitis observed in SpA, whose list includes a number of Th17-associated genes (*CCR6*, *JAK2*, *TYK2*, *STAT3* and *IL23R*; Anderson et al., 2011).

Finally, it is worth reminding that the IL-17 family includes six cytokines. The role of some of them is poorly understood (McGeachy et al., 2019) but they seem central in intestinal immunity: IL-17D has a homeostatic role in the gut (it maintains ILC3 function; Huang et al., 2021), and conversely, IL-17F could promote an inflammation-promoting microbiota, by interfering with the colonisation of Treg-inducing bacteria (Tang et al., 2018), making it a potentially attractive target for pathologic, non-homeostatic, responses. IL-23 is also upregulated in the gut of AS patients (Ciccia et al., 2009), probably secondary molecular events, such as autophagy (Ciccia et al., 2014), and also relevant in CD (Eken et al., 2014).

## Discussion

Experimental data suggesting heterogeneity within the Th17 compartment come largely from mouse studies, where a number of conditioning elements and some molecular networks conferring pathogenic potential have been identified. Whether the majority of these is also relevant in human Th17 is not yet clear. What is increasingly apparent is that Th17 are able to adopt both housekeeping, anti-bacterial gene modules and inflammatory, pathogenic functions, and only the latter would cause immune diseases, such as SpA. The tissue environment is possibly decisive, particularly at barrier sites, where cytokine concentrations or the presence of metabolites can dictate the cell fate. To understand the biology of tissue Th17, careful immune characterisation in steady state and in tissue context will be required. GWAS have discovered associations between genomic loci associated with Th17 function, and the occurrence of AS, providing suggestive hints into pathogenesis (Knight, 2013), but comprehending the functional meaning of the variants is particularly challenging when the genetic association is agnostic of the cell subset and tissue of origin. Since small genetic defects could originate at every phase of cell differentiation or in specific tissues, we believe that combining functional genomics with system immunology will be the next biggest challenge for translational science of immune-mediated diseases.

## Author Contributions

All authors listed have made substantial, direct and intellectual contribution to the work and approved it for publication.

## Funding

Publication fees were supported by the Foundation for Research in Rheumatology (FOREUM) under award number 1016807. The content is solely the responsibility of the authors and does not necessarily represent the official views of the National Institutes of Health.

## Conflict of Interest

The authors declare that the research was conducted in the absence of any commercial or financial relationships that could be construed as a potential conflict of interest.

The handling editor declared a past co-authorship with one of the authors FC.

## Publisher’s Note

All claims expressed in this article are solely those of the authors and do not necessarily represent those of their affiliated organizations, or those of the publisher, the editors and the reviewers. Any product that may be evaluated in this article, or claim that may be made by its manufacturer, is not guaranteed or endorsed by the publisher.
